# A systematic review of the frequency of features of the seven‐point checklist in proven cutaneous melanoma: The importance of change

**DOI:** 10.1002/ski2.295

**Published:** 2023-10-07

**Authors:** Nicola M. Congdon, Christina M. Davis

**Affiliations:** ^1^ University of the West of England Bristol UK

## Abstract

**Background:**

Pigmented skin lesions in human adults can present with several different visible features that may indicate signs of malignancy, particularly melanoma. Patient and clinician awareness of these features can aid the early recognition and melanoma diagnosis improving patient outcomes. The seven‐point checklist (7PCL) is a clinical prediction rule advocated by the National Institute for Health Care Excellence to aid the assessment of pigmented skin lesions in primary care to indicate referral for specialist opinion.

**Objectives:**

Assess the current evidence to establish which features of the 7PC present more frequently, so public education and clinician assessment can be focused to maximise early diagnosis and minimise referrals of benign lesions.

**Methods:**

A systematic review of published evidence identified studies that assessed the seven features of the 7PCL in histologically proven melanomas. Two independent reviewers screened eligible studies and independently extracted data and assessed quality.

**Results:**

112 studies were screened, 20 were assessed in full, seven met the inclusion criteria. 1184 histologically diagnosed melanomas were assessed using the 7PCL. Four studies involved patients assessing 335 melanomas, and three involved clinicians who assessed 849 melanomas. The most common feature identified was a change in size of the lesion, and the least common was inflammation.

**Conclusions:**

The most frequently occurring features of melanoma involve shape, size and colour, however focussing on changes in features, rather than irregularity, is more likely to identify early melanoma and increase the accuracy of referrals.

## Graphical abstract

This review assesses the frequency at which individual features of the seven‐point checklist ‐ used by primary care clinicians to assess pigmented skin lesions for malignancy ‐ occurs in histologically diagnosed melanoma. The results show changes in size, colour and shape were most frequent whilst change in sensation, crusting and oozing and inflammation occurred the least frequently.

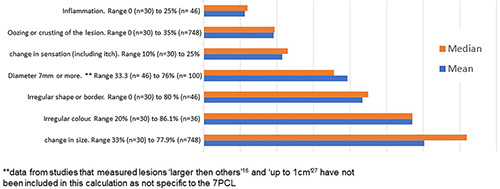

**What is already known about this topic?**

- There are currently 2 lesion recognition tools in frequent use. ABCDE for the public and the seven‐point check list for General practitioners. Some features included in the tools overlap and some have been called into question.
- Studies have assessed the sensitivity and specificity of the tools as a whole, but no systematic review has assessed the frequency that individual features present in histologically proven melanoma.

**What does this study add?**

- The most frequent features seen in biopsy proven melanomas are changes in size, colour and shape. The least frequent features are itch, inflammation, and crusting –also seen in many benign lesions.
- Focussing on changes in features rather than ‘irregular’ features may improve melanoma recognition and reduce the number of benign lesions referred for specialist opinion.

## 1 INTRODUCTION

Melanoma is currently the fifth most common cancer in the UK with around 16 700 people diagnosed in the UK each year.^1^ Even though most melanomas are visible on the skin, diagnoses continue to increase ‐ the World Health Organisation predicts deaths from melanoma to reach 3119 in 2025 in the UK alone.^2^ The earlier melanoma is diagnosed the greater the survival rates,^3^, ^4^ so emphasis must be placed not only on preventative measures but early recognition. Recognition and initial assessment of suspicious skin lesions begin with the naked eye before closer examination with dermoscopy. This systematic review (SR) will focus on naked eye (clinical) assessment.

Clinical prediction rules (CPRs) are tools to guide clinicians' decision‐making.^5^ There are CPRs for clinical and dermoscopic assessment of skin lesions. A 2017 SR found 4 clinical CPR's: ABCD; ABCDE; the seven‐point checklist (7PCL) and the revised/weighted 7PCL.^6^ The ABCD mnemonic (Asymmetry Border; Colour; Diameter) was developed in 1985^7^ and is still used by primary care clinicians in the USA and Australia.^8^, ^9^

The original 7PCL, known as the Glasgow Checklist, was introduced by MacKie^10^ in 1986, following a study demonstrating many patients with melanoma delayed presentation due to lack of information regarding lesion recognition. The checklist was revised in 1989, dividing the features into three major and four minor features.^11^

Since 2015, the National Institute for Health Care Excellence (NICE) advocated the weighted 7PCL for primary care clinicians' assessment of pigmented skin lesions^12^ based on the evidence of two studies. Appendix 1.^13^ The first study prospectively studied 688 people at increased risk of melanoma over 10 years.^14^ Lesions were screened by naked eye examination and dermoscopy, but the study authors centre their conclusion on results from the dermoscopic algorithm version of the 7PCL, which has different criteria based on pattern analysis devised by Argenziano et al.^15^ The second study analysed the original and weighted 7PCL in 1436 lesions in primary care using naked eye examination only, however only 36 lesions proved to be melanoma.^16^ NICE updated its evidence in July 2022 but continues to advocate the 7PCL. The evolution of the 7PCL can be seen in Table 1.

**TABLE 1:** Evolution of the Seven Point. Checklist This table shows the changes in terminology used and the order the features were presented as the checklist evolved.

Author	MacKie 1985^10^	MacKie 1989^11^	MacKie 1990^51^	NICE 2015^12^
Features	Itch	Change in size (major—2 points)	Change in size (major—2 points)	Change in size. (Major—2 points)
	Size (around 1 cm)	Irregular shape (major—2 points)	Change in shape (major—2 points)	Irregular shape or border. (Major—2 points)
	Increasing size	Irregular colour (major—2 points)	Change in colour (major—2 points)	Irregular colour. (Major—2 points)
	Shape (irregular outline)	Largest diameter 7 mm or greater (minor‐ 1 point)	Inflammation (minor‐ 1 point)	Largest diameter 7 mm or more. (Minor‐ 1 point)
	Colour variation (including blue/white tinge and red, white or blue pigmentation)	Inflammation (minor‐ 1 point)	Crusting or bleeding (minor‐ 1 point)	Inflammation. (Minor‐ 1 point)
	Inflammation (inflammatory flare to the edge)	Oozing, crusting or bleeding (minor‐1 point)	Sensory change (minor‐ 1 point)	Oozing or crusting of the lesion. (Minor‐ 1 point)
	Crusting or bleeding (often causing clothes to stick to clothing)	Change in sensation (minor‐ 1 point)	Diameter ≥7 mm (minor‐ 1 point)	Change in sensation (including itch). (Minor‐ 1 point)

*Note*: Lesions scoring 3 or more should be referred for a specialist opinion.

In dermatology, the ABCD/E and 7PCL are both commonly accepted CPRs for assessing pigmented skin lesions and have overlapping features. For this review, only the 7PCL is included as it includes all the features of the ABCDE CPR as well as three unique features as shown in Table 2.

**TABLE 2:** Features of the ABCDE and weighted seven point checklist (7PCL).

ABCDE		Weighted 7PCL
Asymmetry	Overlap	Irregular shape or border (2 pts)
Border	Overlap	Irregular shape or border (2 pts)
Colour	Overlap	Irregular colour (2 pts)
Diameter (greater than 6 mm)	Overlap	Largest diameter 7 mm or more (1 pt)
Evolving	Overlap	Change in size (2 pts)
	Unique	Inflammation (1 pt)
	Unique	Ch ange in sensation (including itch) (1 pt)
	Unique	Oozing or crusting of the lesion (1 pt)

### 1.1 Objectives

Evaluate the frequency that each feature of the 7PCL presents in histologically proven melanomas in human adults and consider if the checklist is a robust CPR to assess pigmented skin lesions in adult humans.

## 2 MATERIALS AND METHODS

### 2.1 Methods

The Preferred Reported Items for SRs and Meta‐Analysis (PRISMA)^17^ was applied to ensure robust, transparent reporting.^18^ The protocol was registered to the International prospective register of SRs (PROSPERO),^19^ registration number CRD42022339359. To minimise errors, data extraction was managed using Covidence,^20^ an online SR management programme.

### 2.2 Eligibility criteria

Eligible studies evaluated adult human patients with clinically suspicious pigmented skin lesions that were: assessed using the 7PCL or included assessment of features of the 7PCL and referred for specialist opinion; assessed by patient and/or clinician; provided histological diagnoses. Excluded studies did not have histological diagnoses; used dermoscopy or other digital assessment tools; assessed acral lesions; included cosmetic lesions; focused on other skin cancers/conditions.

### 2.3 Information sources

A systematic literature search was conducted Feb 2022 of the following databases: CINAHL Plus; AMED ‐ The Allied and Complementary Medicine Database; MEDLINE; ASSIA; Cochrane; Scopus. Prospero was included to search for grey literature and avoid work duplication. A re‐run of the search in March 2023 found no further studies.

### 2.4 Search strategy

The Population, Intervention, Comparison, Outcome (PICO)^21^ model was utilised to develop the research question and search phrases. Appendix 2.

Combinations of the following keywords and Boolean operators were used.

S1—Seven‐point checklist OR revised seven‐point checklist OR weighted seven‐point checklist OR Glasgow checklist.
S2—Primary care OR General Practice OR ambulatory care.
S3—Melanoma.
S4—S1 AND S2 AND S3

No date, geographical location, or language limits were imposed. A hand search of references produced no further studies.

### 2.5 Selection and data collection process

The inclusion and exclusion criteria were applied using Covidence.^20^ Two reviewers (NC, CD) independently screened all articles for inclusion by title and abstract then reviewed all full‐text papers of the included studies to extract data and assess quality. Disagreements were resolved by discussion between reviewers. The inter‐rater reliability of full‐text reviews showed Cohen's Kappa as 0.88344. Data was exported form Covidence to Microsoft Excel for analysis.

### 2.6 Data items

The main data outcome was the frequency of occurrence of each of the 7PCL features in histologically proven melanomas. Other data extracted included country of study; whether the study was retrospective or prospective; the number of lesions examined; the number of melanomas diagnosed (if different from the total number of lesions); if the paper reviewed histologically proven melanoma only, or included lesions histologically reported as benign; who was assessing the lesion—patient or clinician; ethnicity; skin type and gender of participants.

### 2.7 Quality assessment and risk of bias

As none of the included studies involved interventions, highly revered tools such as Cochrane risk‐of‐bias tool could not be adequately applied. All studies in this SR were observational, comprising mostly case reports and case‐control studies, which Oxford Centre for Evidence‐Based Medicine rates as low‐quality evidence.^22^ For this SR, Joanna Briggs Institute (JBI)Critical Appraisal Tools^23^ were utilised, as they included questions assessing risk of bias at study and outcome level, and provided checklists for the included studies.

Critical appraisal results ‐ appendix 3. Questions used by JBI ‐ appendix 4.

### 2.8 Data synthesis

Extracted data is presented as percentages of features seen, with the number of melanomas in the study shown as (*n* = ). Statistical information will be presented where this was provided by the study.

Due to the small number of studies identified and the vast heterogeneity, there was insufficient data for meta‐analysis, so findings will be presented in a narrative synthesis.^24^

## 3 RESULTS

### 3.1 Literature search

**FIGURE 1:**
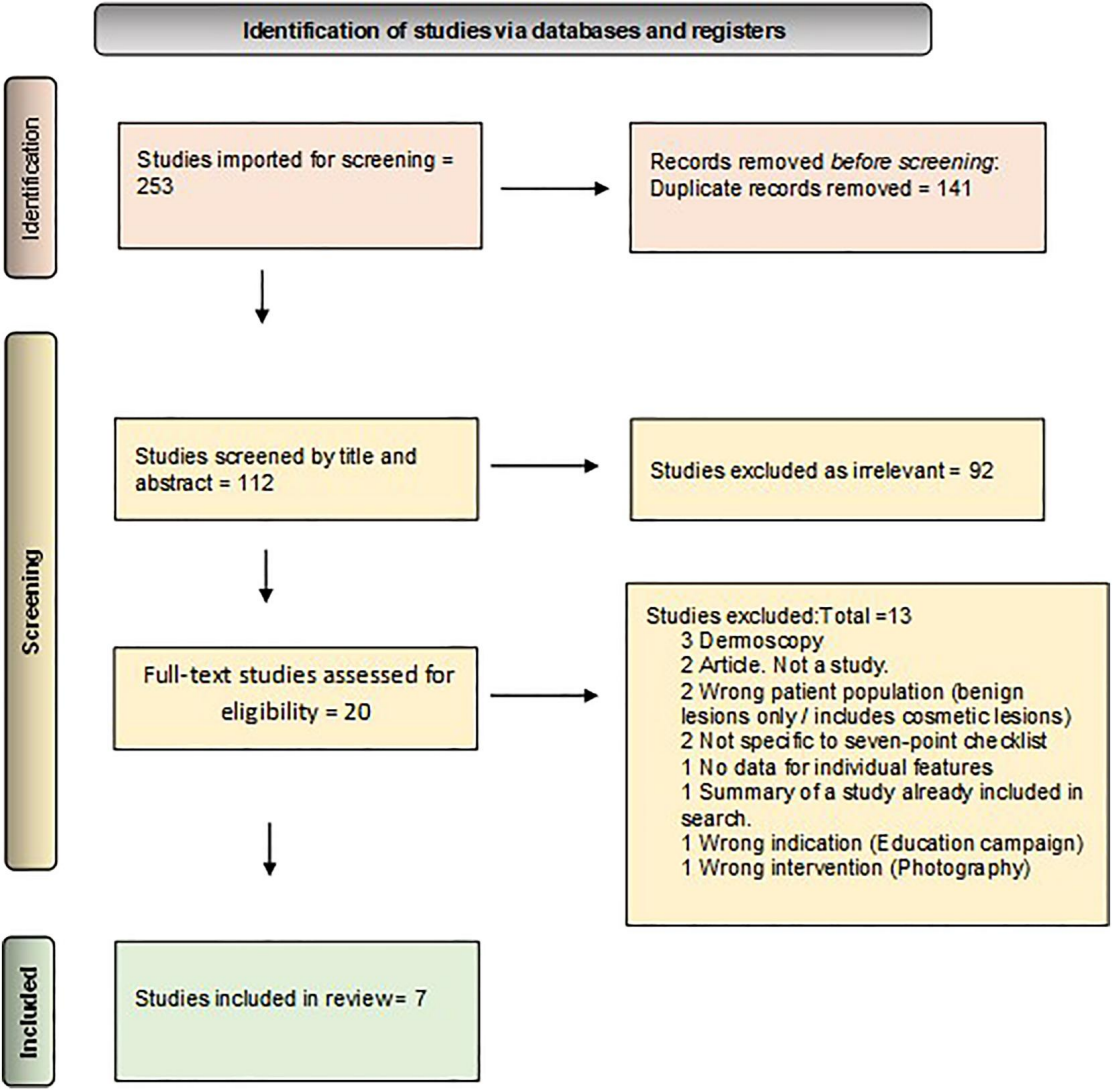
PRISMA flow chart.

Seven studies met the eligibility criteria for inclusion. The PRISMA flow chart (Figure 1) includes the rationale for why some papers that initially met the criteria were ultimately excluded.

### 3.2 Study characteristics

2457 participants were included (mean: 405.85, median: 159) ‐ excluding one study not stating the number of participants, only the number of lesions assessed.^25^ 1494 were females (mean: 249, median: 74.5) and 874 males (mean: 124.86, median: 49). Four studies collated ethnicity^16^, ^26^, ^27^, ^28^ of these, participants were white or Caucasian, which is consistent with typical epidemiology of melanoma.^29^ Only one study provided skin type data.^27^ Age data was challenging to analyse as studies presented this data differently. Results are included in Table 3.

**TABLE 3:** Study characteristics.

Study and aim	Setting and dates	Participant demographics	Study design	Lesions assessed	Lesions assessed by: patient/clinician	Total number of lesions examined	Number of histologically proven melanomas	Findings. Frequency of features are shown greatest to smallest including any statistical analysis where given. Definitions are shown as used in each study	Quality assessment (% of ‘yes’ answers)
Walter 2013^16^ “To validate the original and weighted versions of the 7PCL in the primary care setting.^16^ ^(p.345)^	15 GP practices in the East of England. UK Mar 2008–May 2010	Total = 1182 Female = 758, male = 424. Mean age 44.7 years. 1118 white British. No skin type data provided.	Prospective. Diagnostic validation study.	Melanoma and benign lesions	Clinician	1436	36	Irregular pigmentation: 86.1% (specificity 52.1% *P* < 0.001) Lesion larger than others: 75% (specificity 52.3% *P* 0.001) Change in size of lesion—72.2% (specificity 52.4% *P* 0.004) Irregular border: 69.4% (specificity 66.6% *P* < 0.001) Itch or altered sensation: 25% (specificity 72.3% *P* 0.850) Oozing/crusting of lesion: 19.4% (specificity 90.2% *P* 0.080) Inflammation—16.7% (specificity 91.0% *P* 0.150)	80%
Osborne 1999^30^ “The principal aim of the present study was to investigate the false negative rate of the clinical diagnosis in a large group of histologically diagnosed malignant melanomas in relation to possible predictor variables, with particular reference to the features of the seven‐point check‐list and the seven‐point check‐list score”^30^ ^(p.902)^	Leicester royal infirmary (dept of histopathology). UK 1882–1996	Total = 733 Female = 476, male = 257. Modal age 60 years. No ethnicity or skin type provided	Retrospective. Case series	Melanoma only	Clinician	778	778	Variation pigmentation: 78% (*p* < 0.00001) Changing size: 77.9% (*P* 0.45) Size ≥ 7 mm–76% (*p* < 0.00001) Variation shape: 60% (*p* < 0.00001) Surface oozing/crusting/bleeding: 35% (*P* 0.01) Altered sensation: 22% (*P* 0.20) Inflammation: 12% (*P* 0.00001)	62.5%

Healsmith 1994^25^ “To evaluate the sensitivity and specificity of the revised 7‐point checklist by analysing the features of benign and malignant lesions.”^25^ ^(p.48‐49)^	Pigmented lesion clinic, leicester royal infirmary. UK Nov 1989–Jan 1992	Total not stated, only number of lesions assessed. No ethnicity, skin type or age data provided.	Prospective. Case control	Melanoma and benign lesions	Clinician	165 (65 melanoma and 100 randomly selected benign)	65 consecutively diagnosed MM	Change in colour: 72% Change in size: 72% Change in shape: 45% ≥ 7 mm: 38% Sensory change:23% Ooze/crusting/bleeding: 16% Inflammation: 3%	62.5%
Bränström 2010^26^ “To explore the motives of different patients for seeking medical attention to their pigmented skin lesions.” “To investigate whether other, and perhaps new, features would be mentioned by laymen.”^26 (p.294)^	Department of dermatology and the department of Oncology at the Kerolinska hospital in Stockholm, Sweden.	Total = 90. Female = 48, female with melanoma = 14. Male = 42, Male with melanoma = 16. Melanoma age 30–79 years. Median 59 years. All Caucasian. No skin type data provided.	Prospective. Case control.	Melanoma and benign	Patient	90	30	Started growing: 33% Changed in colour: 20% ≥7 mm: 10% Itching: 10% Changed in shape:0 Bleeding: 0 Inflammation: 0	60%

Du vivier 1991^27^ “To determine: (a) What were the features of the lesion, which alerted the patient to a possible diagnosis of malignant melanoma and (b) how do these figures correspond to the seven‐point checklist.”^27^ (p.344)	Kings College hospital. UK Dates not stated,	Total = 91. Female = 63 Male = 28 77.5% were less than 40 years old All Caucasian. 55% = skin type II.	Prospective. Case series.	Melanoma only	Patient	100	100	Increasing size: 74% Colour variation: 53% Size up to 1 cm: 50% Itch: 24% Abnormal shape: 20% Crusting or bleeding: 18 Inflammation: 11%	62.5%
O'Shea 2017^28^ “The aims of the study were to measure the nature and duration of melanoma symptoms in a group of patients diagnosed with melanoma within the preceding 18 months and to identify the symptoms and barriers associated with a delay in presentation.”^28^ (p.1)	UK Nov 2011	Total 159. Female = 85 Male = 74 Age range 24–90. Mean = 62. 43% older than 65 years old. Ethnicity—all white. No skin type info provided.	Retrospective. Case series	Melanoma only	Patient	159	159	Mole growing bigger: 38.9% Mole changing colour: 32.9% Mole changing shape: 29.5% Mole bleeding or crusting:26.2% Mole itchy: 21.5% *Diameter and inflammation not assessed.	62.5%
Liu 2005^31^ “The purpose of this study was to investigate the observations that patients make that help them to identify melanomas, and to explore how well these Observations correspond to the ABCD(E) rule and the seven‐point checklist"^31^ (p.549)	Victorian melanoma service, the alfred hospital, Victoria, Australia.May 1996–Mar 1998	Total 113. Melanoma = 46, benign = 67 Female = 64. Female with melanoma = 25. Male = 49. Male with melanoma = 21. No ethnicity, skin type or age data provided	Retrospective.Modified case control	Melanoma and benign lesions	Patient	113	46	Change in shape: 80% (OR 6.77 95% CI 0.53–87.09) Change in colour: 57.1% (OR 4.27 95% CI 1.62–11.26) Change in size/new lesion: 54.2% (OR 4.74 95% CI 1.85–12.19) ≥7 mm: 33.3% (OR 0.88 95% CI 0.07–10.6 Sensory changes: 25% (OR 1.07 95% CI 0.21–5.59) Inflammation: 25% (OR 0.52 95% CI 0.04–7.22) Oozing/crusting of: 19.4% (OR 1.76 95% CI 0.27–11.36)	70%

Some participants presented with more than one lesion. 2841 lesions were examined, 1204 were histologically proven melanomas. The number of lesions examined in each study ranged from 90^26^ to 1436,^16^ (mean:401.57, Median: 159). The number of histologically proven melanomas ranged from 30^26^ to 778,^30^ (mean:172, median: 65.)

Studies dated from 1991^27^ to 2017.^28^ Five were conducted in the UK,^16^, ^25^, ^27^, ^28^, ^30^ one in Sweden,^26^ and one in Australia.^31^ There were four prospective studies,^16^, ^25^, ^26^, ^27^ and three retrospective studies.^28^, ^30^, ^31^ Three studies assessed the 7PCL against melanoma only,^27^, ^28^, ^30^ and four included the assessment of melanoma and benign lesions.^16^, ^25^, ^26^, ^31^ In four studies, lesions were assessed by patients^26^, ^27^, ^28^, ^31^ and the remaining three by clinicians.^16^, ^25^, ^30^

Characteristics of the studies, with results of frequency of features identified along with statistical information where available, are presented in Table 3.

### 3.3 Study design

All studies were observational without interventions. Two stated their methodological approach.^16^, ^31^ One was a retrospective modified case‐control,^31^ the other a diagnostic validation study.^16^ The remaining studies were considered to be case‐control studies^25^, ^26^ or case studies.^27^, ^28^, ^30^ Ethical approval was only sought for two studies.^26^, ^31^ Four studies received funding or support from cancer charities.^16^, ^27^, ^28^, ^31^ One study declared competing interests.^16^

### 3.4 Study aims

The study aims can be seen in Table 3. One study did not aim to assess the 7PCL but was included as it provided data for five of the seven features.^28^

### 3.5 Study methodologies

Of the four patient‐assessed studies, one utilised questionnaires^28^ and three used interviews.^26^, ^27^, ^31^ The study using questionnaires^28^ extracted data from a larger study investigating delayed cancer presentation among 15 different cancer types.^32^ Results of this study must be interpreted with caution as the only skin‐related symptom was “Mole Itchiness Rash or red spots,”^32^ (p.584) The questionnaire included a free text section, but results were a low frequency of occurrences for some features compared to other studies. One study interviewing patients asked questions directly related to the 7PCL.^27^ Two studies used open‐ended nondirective questions that were recorded and categorised to features of the 7PCL.^25^, ^31^

Two clinician‐assessed studies were prospective.^16^, ^25^ One examined lesions identified from a previous randomised control trial assessing a novel computerised diagnostic tool.^16^ The other examined lesions presenting to a pigmented skin lesion clinic.^25^ The third retrospectively examined the case notes of patients with histologically proven melanoma from a computerised database over a given period.^30^

Due to the low numbers of studies, results were not divided for subgroup analysis.

## 4 RESULTS ACROSS STUDIES

Results of frequency of occurrence of each feature of the 7PCL, including statistical analysis where provided, can be seen in Table 4.

**TABLE 4:** Results of frequency of occurrence of each feature of the seven‐point checklist (7PCL) including statistical analysis where provided.

Author and year;	Walter 2013^16^	Osborne 1999^30^	Healsmith 1994^25^	Bränström 2010^26^	du vivier 1991^27^	O'Shea 2017^28^	Liu 2005^31^
Number of melanomas;	*n* = 36	*n* = 748	*n* = 65	*n* = 30	*n* = 100	*n* = 100	*n* = 46
Assessed by:	Clinician	Clinician	Clinician	Patient	Patient	Patient	Patient
Change in size %	Change in size of lesion	Changing size	Change in size	Started growing	Increasing size	Mole growing bigger	Change in size/new lesion
	72.2	77.9^a^	72	33	74	38.9	54.2
	Specificity 52.4% *P* 0.004	*P* 0.45					OR 4.74 95% CI 1.85–12.19
Irregular shape or border %	Irregular border	Variation shape	Change in shape	Changed in shape	Abnormal shape	Mole changing shape	Change in shape
	69.4	60^a^	45	0	20	29.5	80
	Specificity 66.6% *p* < 0.001	*p* < 0.00001					OR 6.77 95% CI 0.53–87.09
Irregular colour	Irregular pigmentation	Variation pigmentation	Change in colour	Changed in colour	Colour variation	Mole changing colour	Change in colour
	86.1	78^a^	72	20	53	32.9	57.1
	Specificity 52.1% *p* < 0.001	*p* < 0.00001					OR 4,27 95% CI 1.62–11.26
Largest diameter 7 mm or more %	Lesion larger than others				Size up to 1 cm	Data not provided as this study did not specifically aim to assess the 7PCL.	
	75	76^a^	38	10	50^b^		33.3
	Specificity 52.3% *P* 0.001	*p* < 0.00001					OR 0.88, 95% CI 0.07–10.63
Inflammation %	16.7	12^a^	3	0	11	Data not provided as this study did not specifically aim to assess the 7PCL.	25
	Specificity 91.0%*P* 0.150	*p* < 0.00001					OR 0.52 95% CI 0.04–7.22
Oozing or crusting of the lesion %	Oozing/crusting of lesion	Surface oozing/crusting/bleeding^a^	Ooze/crusting/bleeding	Bleeding	Crusting or bleeding	Mole bleeding or crusting	Crusting or bleeding
	19.4	35^a^	16	0	18	26.2	19.4
	Specificity 90.2% *P* 0.080	*P* 0.01					OR 1.76 95% CI 0.27–11.36
Change in sensation (including itch) %	Itch or altered sensation	Altered sensation	Sensory change	Itching	Itch	Mole itchy	Sensory changes
	25	22^a^	23	10	24	21.5	25
	Specificity 72.3% *P* 0.850	*P* 0.20					OR 1.07 95% CI 0.21–5.59

*Note*: Definitions used by each study are included where they differ from those of the NICE definitions.

^a^ % calculated from numbers given.

^b^ Up to 10 mm (<0.5 = 17. 0.5 – 1 cm = 33, >10 mm = 50).

**FIGURE 2:**
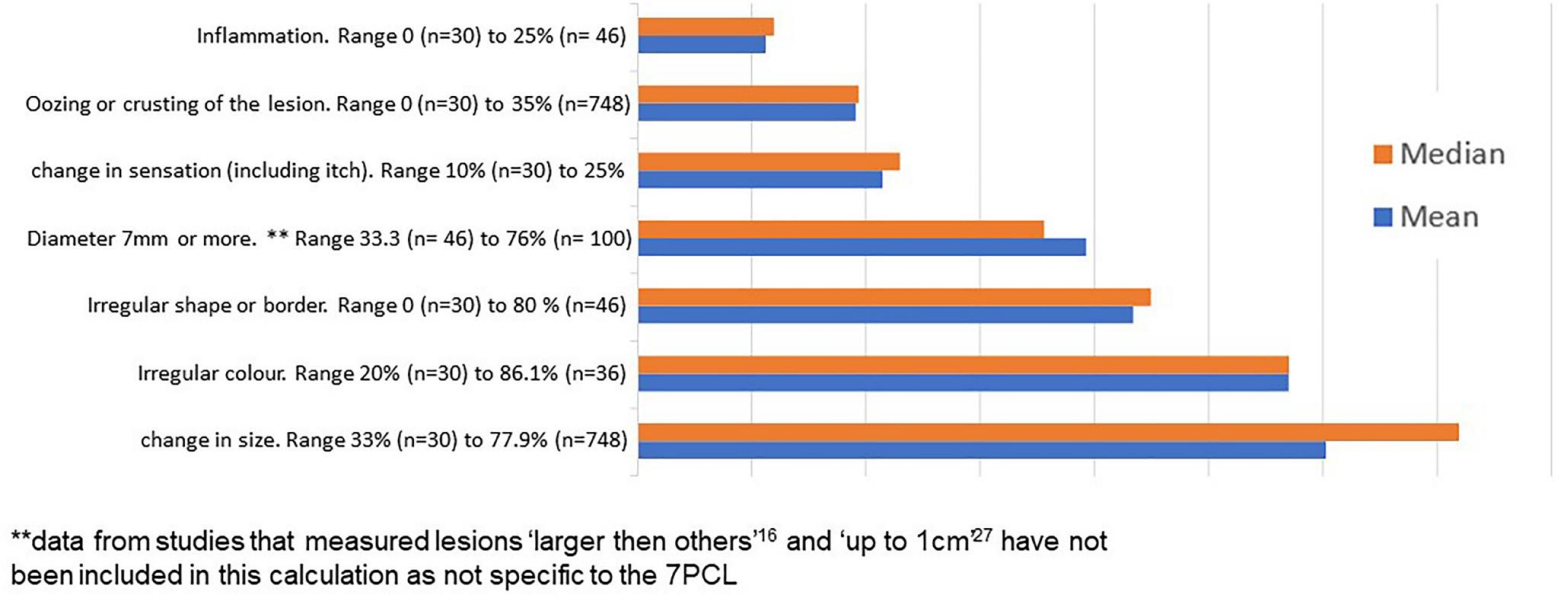
Average frequency of occurrence for each feature of the seven‐point checklist.

Whilst the included studies demonstrated vast heterogeneity and inherent bias, the average frequency of occurrence of the 7PCL was calculated to identify trends in results, which are displayed in Figure 2. No adjustments were made for the study that only included five of the seven features of the 7PCL.^28^ Further interpretation of these results is considered in the following discussion.

## 5 DISCUSSION

This SR found a scarcity of studies assessing the 7PCL including data for the frequency of the individual features. All studies except one were conducted before NICE advocated the 7PCL to assess pigmented skin lesions,^28^ so the terminology did not always match NICE definitions, therefore affecting results. The original 1986 7PCL^10^ preceded the more formal introduction of evidence‐based medicine^33^ and appears based on expert‐led anecdotal evidence and case studies. The book published as an update in 1989^11^ included a reference list, but no direct references to supporting evidence.

The first study that assessed the individual features of the original 7PCL was published 4 years later.^34^ Other studies have since reviewed the 7PCL,^14^, ^35^, ^36^, ^37^ but this is the first known SR assessing the frequency at which features of the 7PCL appear in histologically proven melanoma. Figure 1 includes the reasons these studies were excluded from this SR.

This SR found size change was the most frequently reported feature. Each study, apart from one,^25^ used different definitions to NICE, but were comparable enough to assess a change in lesion size. One study also included ‘new lesions’ in their definition,^31^ but no data was provided to differentiate between a change in size and new lesions.

Colour was the second most frequent feature, however, none of the studies used “irregular colour” as defined by NICE. A common benign mimic of melanoma ‐ seborrhoeic keratoses (SK) ‐ frequently present with irregular colours^38^ and are often a cause of concern to patients. SK can arise from solar lentigos, so do change appearance over a long time period, so the patient lesion history is vital to clinical assessment. Two of the included studies^26^, ^27^ reported that ‘darkening’ or ‘very dark’ lesions were a significant observation among patients. Bränström^26^ concluded ‘very dark’ might be added to future recommendations for identifying melanoma.

Whilst most melanomas are pigmented, amelanotic melanomas are typically red or pink^39^ This less common subtype is difficult to clinically diagnose due to the pigment absence. None of the studies in this SR recording subtypes of melanoma reported amelanotic melanomas, likely due to their rarity and absence of colour.

Irregular shape and border were the third most common feature. All studies used only shape *or* border in their definitions. Another benign mimic of melanoma, solar lentigos, frequently present with irregular shapes or borders,^40^ which may increase benign referrals.

Nodular melanomas ‐ another infrequent melanoma subtype^41^ ‐ tend to stay symmetrical but enlarge rapidly. They are also associated with higher mortality^42^ so it is important that any CPR assessing skin lesions can also identify nodular melanomas.

Diameter ≥7 mm was the fourth most commonly occurring feature, however, only 4 studies specifically reported lesions ≥7 mm. For this review, the studies recording lesions ‘larger than others’^16^ and ‘up to 1 cm’^27^ were removed from the average calculations for diameter, as including them would not accurately reflect the NICE recommended 7PCL of ≥7 mm. The study including “larger than others”^16^ as their diameter definition reported the second highest frequency of occurrence, but this definition may fit better with the ‘ugly duckling’ sign discussed later.

The ABCDE checklist includes diameter of 6 mm or more. One study assessing both checklists, found the ABCDE tool missed 5 melanomas (7.7%) that had changed in size because they were less than 6 mm.^25^ These lesions were picked up by the 7PCL because of the ‘change in size’. Whilst some studies argue diameter of ≤6 mm is a useful parameter,^43^, ^44^ another proposes diameter of ≥7 mm can be called into question.^45^

Definitions used for sensory changes also differ throughout the studies. One study included ‘itch’ and ‘altered sensation’ in their definition.^16^ Three used only itch; in their definition^26^, ^27^, ^28^ whilst the others referred to sensory changes^25^, ^31^ or altered sensations.^16^, ^30^

The original 7PCL booklet states itch is a common feature of malignant and benign lesions,^10^ however, a study of biopsy‐proven skin cancers and SK in elderly males found only 5.3% (*n* = 39) of patients with melanoma reported itch, compared to 25.5% (*n* = 224) of patients with SK.^46^ du Vivier et al.^27^ also observed many benign lesions were referred because of itch alone.

Inflammation definitions were consistent with NICE. MacKie describes inflammation as “a red inflammatory flare around the edge”^10^ (p, 6) of a lesion not seen in benign lesions. Conversely, du Viver et al.^27^ noted inflammation was a common reason for referral of benign lesions.

When assessing oozing and crusting, ‘crusting’ was used in all definitions except one that used only ‘bleeding’.^26^ Three studies included oozing^16^, ^25^, ^30^ and two also included bleeding.^25^, ^30^ ^.^ MacKie reasoned bleeding is “very common”^10^ (p, 6) in early melanomas. Other studies argue bleeding (and itch) are usually late signs of malignancy.^26^, ^47^ The National Cancer Institute supports this,^48^ yet the NICE advocated 7PCL does not include bleeding and only assigns one point to oozing and crusting.

Throughout these studies, change is persistently regarded as the most significant feature of malignancy in pigmented skin lesions. Osborne reported 90% of lesions examined reported a history of change.^30^ A study examining patients' use of the ABCD rule suggests using only ‘evolution’ in public campaigns,^49^ an opinion previously argued by Weinstock et al.^50^

MacKie et al. incorporated change into the 7PCL in 1990,^51^ yet the NICE version of the 7PCL only includes change in size. Shape, border, and colour are assessed as ‘irregular’. This may explain the large number of benign lesions referred for specialist opinion such as solar lentigos that can present with irregular shapes or borders,^40^ and SK, which often have irregular colours, can itch, bleed, and become easily inflamed.^52^

Whilst change is key to identifying melanoma, patients have expressed difficulty in recalling how lesions looked previously.^53^, ^54^ To help identify changes, patients are encouraged to photograph lesions of concern and engage the assistance of a friend or relative to assist self‐monitoring of difficult‐to‐see areas. A study of skin examination in partnerships compared to solo examinations, found patients in partnerships were more likely to engage in skin examination with greater efficiency.^54^ Smartphone apps have also been developed to assist the public in monitoring skin lesions,^55^ however many older patients who are at higher risk of melanoma^56^ do not own smartphones and the accuracy of apps is debatable.^57^, ^58^

Patients are also educated observe for new lesions. O’Shea et al.^28^ reported change in pre‐existing moles in 76% of participants. Conversely, du Vivier et al.^27^ found ¾ of the lesions assessed were new. A meta‐analysis of the prevalence and implications of nevus‐associated melanoma, found 70.9% of melanomas developed as new lesions.^59^ ‘New’ lesions are not currently a feature of the 7PCL.

Patients are also advised to recognise lesions that look different from the rest, often referred to as ‘ugly ducklings’. Walters assessment of diameter as “larger than others” can be viewed as differentiating lesions from others. Liu^31^ observed that some participants commented on ‘ugly’ lesions, but Bränström^26^ believes these features are subjective. In clinical practice it is usually the lesions that stand out as different from the rest that are examined more closely with dermoscopy.

This review has focused on the clinical examination of pigmented lesions. After a suspicious lesion has been detected by the naked eye, dermoscopy can aid making a clinical diagnosis. While some primary care clinicians use dermoscopy to assess pigmented skin lesions, it requires training and regular practice to maintain competence, and is thus not available for use by the public. It is therefore fundamental that any CPR is reliable and robust to help identify early melanoma, something which can improve the quality of life of patients and result in cost savings.^4^ The findings of this SR may influence the revision of the current 7PCL or the development of a new CPR to assist in the assessment of pigmented skin lesions by both the public and primary care clinicians.

### 5.1 Strengths and limitations of review

This review was limited to a small number of observational studies without interventions. Including ‘signs’ and ‘symptoms’ of melanoma in the search criteria may have found further studies examining features of melanoma that could be aligned to features of the 7PCL.

Only features of the 7PCL were included in this review on the basis that it encompassed the features of the ABCDE CPR, however, some studies contend the two CPR's differ in their ability to identify melanoma.^26^, ^31^, ^37^ A SR of both CPR's also concluded differences between them,^60^ however, this was based on the overall CPR's, rather than analysis of individual features.

Whilst averages were used to identify trends in results, heterogeneity between study methods and varying definitions for features of the 7PCL used means these findings should be interpreted with caution. Due to the limited studies available, only the frequency of presentation of features has been reviewed, while this may assist in the assessment of pigmented lesions, further studies providing sensitivities and specificities for each feature would be more likely to minimise referral of some benign lesions, without failing to identify suspicious lesions for specialist referral.

## 6 CONCLUSION

This SR identified the most frequently occurring features of melanoma involve the shape size and colour, however a change in these features are more likely to indicate melanoma than ‘irregular’ features as defined by the 7PCL advocated by NICE. Features involving sensation, oozing, crusting, and inflammation present less frequently and are commonly found in benign mimics of melanoma. They can also be signs of advanced melanomas, so require serious consideration when assessing lesions using the 7PCL. Further studies designed to assess the sensitivities and specificities of each feature of the 7PCL would provide better guidance for melanoma identification and may guide revision of the 7PCL or development of a new CPR for clinical melanoma recognition.

## CONFLICT OF INTEREST STATEMENT

None to declare.

## AUTHOR CONTRIBUTIONS

**Nicola M. Congdon**: Conceptualisation (lead); Data curation (equal); Formal analysis (lead); Validation (lead); Writing – original draft (lead); Writing – review & editing (lead). **Christina M. Davis**: Conceptualisation (supporting); Data curation (equal); Formal analysis (supporting); Validation (supporting); Writing – review & editing (supporting)

## ETHICS STATEMENT

Not applicable.

## Supporting information

Supporting Information S1

## Data Availability

The data that support the findings of this study are available from the corresponding author upon reasonable request.
