# Supplementary material for: A systematic review of the frequency of features of the seven‐point checklist in proven cutaneous melanoma: The importance of change
Source: Skin Health Dis. 2023 Oct 7;3(6):e295. doi: 10.1002/ski2.295 (PMC10690704; doi:10.1002/ski2.295)
Supplement: Supplementary file 1 — Supporting Information S1 [file SKI2-3-e295-s001.docx]

### Appendix 1. Evidence on the ABCD and 7-point checklists | Supporting evidence | Melanoma and ... Page 1 of 1^13^

Melanoma and pigmented lesions:

Evidence on the ABCD and 7-point checklists

Last revised in March 2017

A number of studies have looked at different methods of diagnosing pigmented lesions but many have limitations for application in a primary care setting, such as Dermoscopy which is not commonly practised. The sensitivities and speci cities of the 7-point checklist in comparison to the ABCD(E) rule has varied between studies, with the latter method still being commonly used in North America and Australia. The

Weighted 7-point checklist is has been recommended by NICE since 2015 for practitioners in primary care in the UK to differentiate between benign lesions and suspected melanoma [NICE, 2015b (/topics/melanoma-pigmented-lesions/references/)].

- A non-comparative study prospectively assessed the sensitivity, speci5city, and diagnostic accuracy of the 7-point checklist [Haenssle et al, 2010 (/topics/melanoma-pigmented-lesions/references/)].

◦ Screening by naked-eye examination, the dermatoscopic 7-point checklist, and digital dermatoscopy was carried out over a period of 10 years on 688 people at increased risk of melanoma.

◦ Of the 127 melanomas detected, 50 were melanoma in situ. In total 79 ful5lled the 7-point checklist threshold of 3 or more points (62% sensitivity, less than in retrospective studies). Speci5city was 97% (more than in retrospective studies).

◦ The sensitivity was increased by the use of complementary information

(such as history of the lesion, differences from the person's other moles [ugly duckling sign], and dynamic changes).

- [Walter, 2013 (/topics/melanoma-pigmentedlesions/references/)] analysed the use of the Original 7-point checklist with the Weighted 7-point checklist in primary care

◦ 1580 lesions on 1297 participants were analysed in general practice, showing that both variations performed well in the early identi5cation of melanoma (Comparing lesions scoring 3 points or more from the Original 7-point checklist (sensitivity 80.6% and speci5city 61.7%) and the Weighted 7-point checklist (sensitivity 91.7% and speci5city 33.1%).

◦ The term 'irregular border' was noted to be the most important feature, showing a sensitivity of 69.4% and speci5city 66.6% alone.

◦ The study concluded that a potential cut-off score of 4 (rather than 3) may improve speci5city for lesions without reducing the sensitivity, although this should be assessed further.

© NICE (National Institute for Health and Care Excellence) 2021. All rights reserved. Subject to Notice of rights

### Appendix 2. PICO

| **P** | **P**atient, **P**opulation, or **P**roblem | How would I describe a group of patients similar to mine? | Human adults with pigmented skin lesions presenting for medical review / diagnosed with melanoma. | Frequency of individual features of the Seven Point Checklist in histologically diagnosed cutaneous melanoma |
| --- | --- | --- | --- | --- |
| **I** | **I**ntervention, Prognostic Factor, or Exposure | Which main intervention, prognostic factor, or exposure am I considering? | 7 point check list as recommended by NICE. |  |
| **C** | **C**omparison or Intervention (if appropriate) | What is the main alternative to compare with the intervention? | NA |  |
| **O** | **O**utcome you would like to measure or achieve | What can I hope to accomplish, measure, improve, or affect? | Frequency of occurrence of individual features of the 7PCL in histologically proven melanoma. |  |
|  | What **T**ype of question are you asking? | Diagnosis, Etiology /Harm, Therapy, Prognosis, Prevention | Etiology. |  |
|  | Type of **S**tudy you want to find | What would be the best study design/methodology? | Any. |  |

**Inclusions:**

Adults with pigmented skin lesions assessed using the 7PCL or included assessment of features of the 7PCL and referred for specialist opinion; assessed by patient and/or clinician; provided histological diagnoses.

**Exclusions:**

Excluded studies did not have histological diagnoses; used dermoscopy or other digital assessment tools; assessed acral lesions; included cosmetic lesions; focused on other skin cancers/conditions.

**Appendix 3. Joanna Briggs Institute critical appraisal results.**

| **Reference** | **JBI tool used** | **Q1** | **Q2** | **Q3** | **Q4** | **Q5** | **Q6** | **Q7** | **Q8** | **Q9** | **Q10** | **Results**  **(% of yes answers)** | **Include/Exclude** |
| --- | --- | --- | --- | --- | --- | --- | --- | --- | --- | --- | --- | --- | --- |
| Walter et al.^16^ | Diagnostic validation | Yes | Yes | Yes | NA | Yes | Yes | Yes | NA | Yes | Yes | 80% | Include |
| Liu et al. ^31^ | Case control | Yes | Yes | Yes | Yes | Yes | NA | NA | Yes | NA | Yes | 70% | Include |
| Healsmith et al.^25^ | Case Control | Yes | Yes | Yes | Yes | Yes | NA | NA | Yes | NA | No | 60% | Include |
| Bränström et al.^26^ | Case Control | Yes | Yes | Yes | Yes | Yes | NA | NA | Yes | NA | No | 60% | Include |
| Osborne et al. ^30^ | Case report | Yes | NA | Yes | Yes | Yes | NA | NA | Yes | - | - | 62.5% | Include |
| du Vivier et al.^27^ | Case report | Yes | NA | Yes | Yes | Yes | NA | NA | Yes | - | - | 62.5% | Include |
| O’Shea et al. ^28^ | Case report | Yes | NA | Yes | Yes | Yes | NA | NA | Yes | - | - | 62.5% | Include |

### Appendix 4 – Joanna Briggs Institute Appraisal tool questions.

|  | **Q1** | **Q2** | **Q3** | **Q4** | **Q5** | **Q6** | **Q7** | **Q8** | **Q9** | **Q10** |
| --- | --- | --- | --- | --- | --- | --- | --- | --- | --- | --- |
| Diagnostic validation | Was a consecutive or random sample of patients enrolled? | Was a case control design avoided? | Did the study avoid inappropriate exclusions? | Were the index test results interpreted without knowledge of the results of the reference standard? | If a threshold was used, was it pre-specified? | Is the reference standard likely to correctly classify the target condition? | Were the reference standard results interpreted without knowledge of the results of the index test? | Was there an appropriate interval between index test and reference standard? | Did all patients receive the same reference standard? | Were all patients included in the analysis? |
| Case control | Were the groups comparable other than the presence of disease in cases or the absence of disease in controls? | Were cases and controls matched appropriately? | Were the same criteria used for identification of cases and controls? | Was exposure measured in a standard, valid and reliable way? | Was exposure measured in the same way for cases and controls? | Were confounding factors identified? | Were strategies to deal with confounding factors stated? | Were outcomes assessed in a standard, valid and reliable way for cases and controls? | Was the exposure period of interest long enough to be meaningful? | Was appropriate statistical analysis used? |
| Case report | Were patient’s demographic characteristics clearly described? | Was the patient’s history clearly described and presented as a timeline? | Was the current clinical condition of the patient on presentation clearly described? | Were diagnostic tests or assessment methods and the results clearly described? | Was the intervention(s) or treatment procedure(s) clearly described? | Was the post-intervention clinical condition clearly described? | Were adverse events (harms) or unanticipated events identified and described? | Does the case report provide takeaway lessons? | NA | NA |
